# Anterior chamber fibrinoid syndrome after cataract extraction in a patient on ibrutinib for B-cell chronic lymphocytic leukemia: a case report and review of the literature

**DOI:** 10.1186/s13256-018-1822-9

**Published:** 2018-11-16

**Authors:** Anton M. Kolomeyer, Christopher K. Hwang, Benjamin J. Kim

**Affiliations:** Department of Ophthalmology, Scheie Eye Institute, University of Pennsylvania, 51 N. 39th St, Philadelphia, PA 19104 USA

**Keywords:** Fibrinoid syndrome, Fibrinoid reaction, Anterior chamber, Phacoemulsification, Ibrutinib, Chronic lymphocytic leukemia

## Abstract

**Background:**

Ibrutinib is a tyrosine kinase inhibitor commonly used in patients with chronic lymphocytic leukemia. Based on the published literature, it has a very sound ophthalmologic safety profile. In the following, we describe a case of anterior chamber fibrinoid syndrome in a patient on ibrutinib for B-cell chronic lymphocytic leukemia after uncomplicated cataract extraction.

**Case presentation:**

A 75-year-old white man with B-cell chronic lymphocytic leukemia on ibrutinib therapy and without significant past ocular history presented 1 day after uncomplicated phacoemulsification with in-the-bag intraocular lens implantation with multiple, discrete, pigmented cords in the anterior chamber. His vision was 20/100 and intraocular pressure was 43 mmHg. There was no hypopyon, hyphema, or cellular reaction. The dilated fundus examination was unremarkable. He was diagnosed as having fibrinoid syndrome and started on topical prednisolone, brimonidine, timolol-dorzolamide, and orally administered acetazolamide. Within 2 weeks, the fibrin cords disappeared completely, vision improved to 20/30, and the intraocular pressure normalized off all medications.

**Conclusions:**

The precise etiology of fibrinoid syndrome remains unclear. This is the first case of fibrinoid syndrome in a patient on ibrutinib, which is known to cross the blood–brain barrier and induce intraocular changes. It is important to differentiate this syndrome from toxic anterior segment syndrome and endophthalmitis, and to initiate appropriate treatment. The fibrin bands tend to be exquisitely sensitive to topical steroids and to resolve within a few weeks without sequelae.

## Background

Fibrinoid syndrome was a term first used by Sebestyen in 1982 to refer to patients with proliferative diabetic retinopathy (PDR) who developed thick transvitreal or retropupillary fibrin bands or cords in the vitreous cavity after undergoing multiple surgical procedures for diabetic sequelae [1]. It may result in complications and poor visual outcome despite the use of topical and systemic steroids. A similar constellation of clinical findings was attributed to extensive cryotherapy by Machemer in 1975 and to hypotony by Schepens in 1981 [2, 3].

The signs of fibrin or fibrinoid reaction can range from the presence of a few fibrin strands to a dense pupillary membrane in the anterior chamber [4]. It is most commonly associated with diabetes, pseudoexfoliation, glaucoma, and extracapsular cataract extraction (ECCE) [5]. It typically presents within 1–2 weeks after surgery, lasts up to 3–4 weeks, and resolves completely without permanent sequelae after initiation of topical steroid therapy.

Ibrutinib is an irreversible inhibitor of Bruton tyrosine kinase that is commonly used in the management of patients with chronic lymphocytic leukemia (CLL) [6]. It is known to cross the blood–brain barrier (BBB) and is able to gain access to and affect the anterior chamber based on preclinical studies and clinical reports [7–10].

## Case presentation

**Fig. 1:**
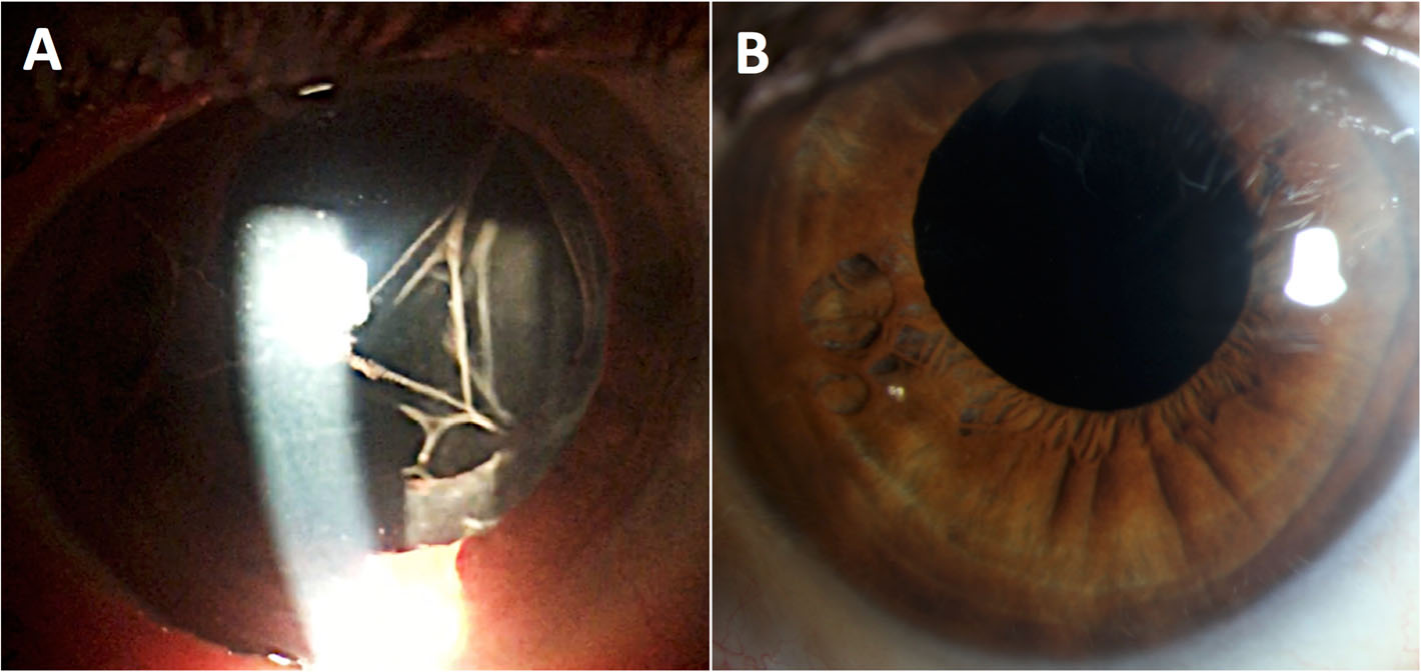
**a** Thick, rope-like fibrin cords in the anterior chamber 1 day after uncomplicated phacoemulsification with in-the-bag intraocular lens implantation. **b** The cords resolved 2 weeks after topical steroid administration

A 75-year-old white man presented 1 day after uncomplicated phacoemulsification and in-the-bag intraocular lens (IOL) implantation with multiple, intertwined, discrete, pigmented cords in the anterior chamber (Fig. 1a). The fellow eye was phakic with best-corrected vision of 20/30 and had not undergone any prior surgeries/procedures. He did not have a history of diabetes, glaucoma, uveitis, trauma, or other intraocular surgery. Past medical history was significant for atrial fibrillation, Raynaud’s syndrome, and B-cell CLL previously treated initially with rituximab and chlorambucil, and more recently with ibrutinib for 6 months prior to cataract extraction. The lens had 2–3+ nuclear sclerosis without pseudoexfoliation or phacodonesis, and did not require mechanical pupil expansion. A retrobulbar block of 2% lidocaine and 0.75% Marcaine (bupivacaine) was administered preoperatively. No intracameral or intravitreal medications were used. At the end of the case, dexamethasone and cefazolin were applied to the ocular surface.

At presentation, his vision was 20/100 and intraocular pressure (IOP) was 43 mmHg. There was no hypopyon, hyphema, significant corneal edema, or cellular reaction. The dilated fundus examination was unremarkable. Fibrinoid syndrome was suspected. He was started on topical prednisolone every 2–3 hours, brimonidine three times per day, timolol-dorzolamide two times per day, and orally administered acetazolamide. Within 2 weeks, the cords disappeared completely (Fig. 1b), vision improved to 20/30, and the IOP normalized off all medications.

## Discussion

Fibrinoid syndrome was originally described in patients with PDR who underwent procedures such as pars plana vitrectomy (PPV), scleral buckling, and lensectomy [1]. The fibrin developed on the retinal surface or behind the iris 2–14 days after surgery and eventually progressed into a vitreous fibrous mass that caused tractional retinal detachment, iris neovascularization, and neovascular glaucoma. Despite oral and topical steroids, 9/15 (60%) patients progressed to no light perception vision. Patients with preoperative retinal detachment and those requiring multiple surgical procedures were significantly more likely to develop this syndrome.

Additional studies limited to the posterior segment described a “fibrinoid”, “web-like”, or “cobweb-like” response as thick vitreous cords that are responsive to steroids and resolve without sequelae [11–13].

A vitreous “web-like inflammatory response” was also noted in a patient with diabetic retinopathy who underwent PPV, membrane peel, and bevacizumab injection for an epiretinal membrane [12]. The authors postulated that this might have been a toxic anterior segment syndrome (TASS)-like or non-infectious endophthalmitis-like reaction to bevacizumab.

Thanos and coworkers described a patient is his 50s with diabetes who underwent uneventful PPV for non-clearing vitreous hemorrhage (NCVH) in the right eye and then developed “cobweb-like tan strands” and elevated IOP after PPV with endolaser for NCVH in the left eye [13]. Similar to our patient, the strands were responsive to frequent topical steroid drops and the elevated IOP was successfully managed with IOP-lowering medications. The cause of IOP elevation in both of these cases is probably the exuberant inflammatory response of fibrinoid syndrome; however, especially for anterior segment surgery, other etiologies such as insufficient viscoelastic removal should be considered as well.

Luo *et al.* reported on seven patients with PDR who developed a “transvitreal fibrinoid response” 1 day after PPV for diabetic sequelae [11]. Mean time to band resolution while on topical steroid therapy was 8.75 days, and correlated with vitreous band density.

In contrast to the thick, rope-like cords characteristically seen in the posterior segment, anterior segment “fibrinoid”, “fibrin”, or “fibrinous” reaction designates a more common finding that has a spectrum ranging from a few thin strands to a thick pupillary plaque [4, 5, 14].

Walinder and colleagues noted a “fibrinoid reaction” in 11–17% of patients undergoing ECCE with IOL placement [4]. It occurred 1–6 days after surgery, lasted up to 3 weeks, and morphologically ranged from a few strands to a dense pupillary membrane. There was no hypopyon, keratic precipitates, or vitreous reaction. Few patients developed posterior synechiae. The authors identified a significant association between fibrinoid reaction, pseudoexfoliation syndrome, and capsular glaucoma.

Baltatzis and coworkers determined the incidence of “fibrin reaction” in patients with diabetes without retinopathy (*n* = 102), primary open angle glaucoma with prior surgery (*n* = 78), and pseudoexfoliation without glaucoma (*n* = 43) as 13.7%, 44.8%, and 27.9%, respectively [5]. Similarly to Walinder *et al*. [4], the fibrin appeared between 3 and 12 days after surgery, was usually located in the pupillary area, resolved after a few weeks, and responded well to steroid therapy.

Jabbur described a patient with chronic leukemia and thrombocytopenia on aminocaproic acid who developed an anterior chamber “fibrinous reaction” 1 day after phacoemulsification with IOL implantation [14]. There was no hypopyon, hyphema, or cellular reaction. Fibrin accumulation was attributed to the effects of aminocaproic acid on the blood–aqueous barrier (BAB). By a similar mechanism, mannitol has been shown to increase anterior chamber flare in young adults as well as older adults undergoing cataract extraction [15].

Although the causative mechanism(s) of fibrinoid syndrome/reaction remains unclear, most commonly proposed etiologies include endothelial dysfunction and increased vascular inflammation/permeability [1]. Idiosyncratic, sterile, non-infectious responses to intravitreal medication administration and pharmacologic alteration of the BAB have been implicated as well [12, 14, 15]. In our patient, we propose that ibrutinib may have contributed to the development of anterior chamber fibrinoid syndrome via several mechanisms.

Our patient is unusual in that his clinical findings developed after uncomplicated phacoemulsification with IOL implantation without any of the previously reported predisposing risk factors for fibrinoid syndrome (that is, multiple surgical procedures for diabetic complications) or fibrinoid reaction (that is, history of diabetes, pseudoexfoliation, glaucoma, or uveitis). On morphological examination, he had thick rope-like fibrin cords similar to those found in the posterior segment [1, 11–13] rather than the thin strands or plaque typical of the anterior segment [4, 5, 14]. Therefore, we consider our case as an uncommon anterior chamber fibrinoid syndrome as opposed to the more typical anterior chamber fibrin reaction.

A previously published case of “fibrinous reaction” in a patient on aminocaproic acid [14] as well as reports of anterior chamber flare after mannitol administration [15] motivated us to closely examine the possibility of a pharmacologic cause for the clinical presentation in our patient. We identified ibrutinib as the most likely medication responsible as it has been shown to cross the BBB and induce anterior chamber changes [7, 8]. In the RESONATE multicenter phase 3 study of ~ 400 patients with relapsed or refractory CLL or small lymphocytic lymphoma on ibrutinib versus ofatumumab (anti-CD20 antibody), 10% of patients on ibrutinib developed blurred vision and 3% developed cataracts [10]. The authors of the study warned that “longer exposure may be associated with an increased risk” of such adverse effects. In addition, Neffendorf and colleagues reported “peculiar lens opacities” in an 80-year-old patient on ibrutinib for 6 months and suggested “precautionary baseline and repeated visual-acuity testing” in these patients [9]. Based on the above, it is reasonable to assume that, in addition to crossing the BBB, ibrutinib is able to cross the BAB and the blood–retina barrier.

In addition to inducing anterior chamber changes and the visual effects described above, we propose that ibrutinib’s pharmacologic mechanism of action may have contributed to the formation of thick fibrin cords in our patient. Platelet dysfunction is a well-documented side effect of ibrutinib [16]. Platelets are major producers of plasminogen activator inhibitor-1, which is an anti-fibrinolytic protein [17]. Fibrinolysis is an important component of fibrin resolution, and intracameral fibrinolytic agents such as tissue plasminogen activator (TPA) have been used to manage exuberant fibrin reaction in the anterior chamber [18]. Therefore, it is possible that ibrutinib-associated platelet dysfunction could lead to fibrinolysis inhibition ultimately resulting in our patient’s presentation. Interestingly, TPA is produced and localized to the uveal microvasculature, corneal endothelium, and trabecular meshwork [19]. Thus, ibrutinib could have contributed to our patient’s presentation through a direct intraocular effect via TPA dysregulation and fibrinolysis inhibition.

Finally, although ibrutinib has a strong safety profile and is generally well tolerated, a few patients developed neutrophilic panniculitis, which is an inflammatory infiltration of the subcutaneous adipose tissue [20]. An exuberant adaptive immune response against a novel hapten epitope (“drug-induced immune modulation”) was proposed to explain the pathogenesis of this rare finding. The majority of patients were successfully treated with low-dose systemic corticosteroids. It is intriguing to think that this may represent an idiosyncratic proinflammatory reaction, and if a similar mechanism could have contributed to the findings in our patient.

## Conclusions

The precise etiology of fibrinoid syndrome, especially of the anterior segment, remains unknown; however, it can be safely assumed that it is multifactorial. We present a case of anterior segment fibrinoid syndrome in a patient on ibrutinib for B-cell CLL after uncomplicated cataract extraction, and propose several mechanisms by which this medication could have caused this unusual clinical entity.

It is important to recognize fibrinoid syndrome in order to differentiate it from TASS and endophthalmitis, and to initiate appropriate treatment. The presentation is characterized by lack of pain, exuberant postoperative swelling/redness, significant corneal edema, or anterior chamber/vitreous reaction. Prompt initiation of anti-inflammatory therapy and close follow-up is paramount as the fibrin bands are exquisitely sensitive to topical steroids and resolve within a few weeks without permanent sequelae.

## Abbreviations

BAB: Blood–aqueous barrier
BBB: Blood–brain barrier
CLL: Chronic lymphocytic leukemia
ECCE: Extracapsular cataract extraction
IOL: Intraocular lens
IOP: Intraocular pressure
NCVH: Non-clearing vitreous hemorrhage
PDR: Proliferative diabetic retinopathy
PPV: Pars plana vitrectomy
TASS: Toxic anterior segment syndrome
TPA: Tissue plasminogen activator

## References

[1] Sebestyen JG. Fibrinoid syndrome: a severe complication of vitrectomy surgery in diabetics. Ann Ophthalmol. 1982;14:853–6. 7181348

[2] Schepens CL. Clinical and research aspects of subtotal open-sky vitrectomy. XXXVII Edward Jackson Memorial Lecture. Am J Ophthalmol. 1981;91:143–71. 10.1016/0002-9394(81)90168-9 6970523

[3] Machemer R. Vitrectomy: A Pars-Plana Approach. New York: Grune and Stratton Inc; 1975. p. 118.

[4] Walinder PE, Olivius EO, Nordell SI, Thorburn WE. Fibrinoid reaction after extracapsular cataract extraction and relationship to exfoliation syndrome. J Cataract Refract Surg. 1989;15:526–30. 10.1016/s0886-3350(89)80109-9 2810086

[5] Baltatzis S, Georgopoulos G, Theodossiadis P. Fibrin reaction after extracapsular cataract extraction: a statistical evaluation. Eur J Ophthalmol. 1993;3:95–7. 10.1177/112067219300300208 8353437

[6] Wiestner A. Emerging role of kinase-targeted strategies in chronic lymphocytic leukemia. Hematol Am Soc Hematol Educ Program. 2012;2012:88–96. 10.1182/asheducation-2012.1.88 23233565

[7] Keller DA, Brennan RJ, Leach KL. Antitargets and Drug Safety. In: Urban L, Patel V, Vaz RJ, editors. Antitargets and Drug Safety. Weinheim: WILEY-VCN; 2015. p. 387–8.

[8] Gauthier AC, Nguyen A, Munday WR, Xu ML, Materin MA. Anterior chamber non-Hodgkin lymphoma of the iris masquerading as uveitis-glaucoma-hyphema syndrome. Ocular Oncol Pathol. 2016;2:230–3. 10.1159/000445119 PMC5091236 27843901

[9] Neffendorf JE, Gout I, Hildebrand GD. Ibrutinib in relapsed chronic lymphocytic leukemia. N Engl J Med. 2013;369:1277. 10.1056/NEJMc1309710 24066759

[10] Byrd JC, Brown JR, O'Brien S, *et al.* Ibrutinib versus ofatumumab in previously treated chronic lymphoid leukemia. N Engl J Med. 2014;371:213–23. 10.1056/NEJMoa1400376 PMC4134521 24881631

[11] Luo C, Ruby A, Neuwelt M, Williams GA. Transvitreal fibrinoid pseudoendophthalmitis after diabetic vitrectomy. Retina. 2013;33:2069–74. 10.1097/IAE.0b013e31828abe67 23609127

[12] Chiang A, Reddy S, Tsui I, Hubschman JP. Vitreous web after pars plana vitrectomy and bevacizumab with fluid-air exchange. Semin Ophthalmol. 2011;26:25–7. 10.3109/08820538.2010.541316 21275601

[13] Thanos A, Modjtahedi BS, Eliott D. An Unexpected Postvitrectomy Course. JAMA Ophthalmol. 2016;134:937–8. 10.1001/jamaophthalmol.2015.6117 27254533

[14] Jabbur NS. Excessive fibrin after cataract surgery associated with aminocaproic acid use. J Cataract Refract Surg. 2003;29:1636–7. 10.1016/s0886-3350(03)00075-0 12954320

[15] Miyake K, Miyake Y, Maekubo K. Increased aqueous flare as a result of a therapeutic dose of mannitol in humans. Graefes Arch Clin Exp Ophthalmol. 1992;230:115–8. 10.1007/BF00164647 1577289

[16] Levade M, David E, Garcia C, *et al.* Ibrutinib treatment affects collagen and von Willebrand factor-dependent platelet functions. Blood. 2014;124:3991–5. 10.1182/blood-2014-06-583294 25305202

[17] Brogren H, Karlsson L, Andersson M, Wang L, Erlinge D, Jern S. Platelets synthesize large amounts of active plasminogen activator inhibitor 1. Blood. 2004;104:3943–8. 10.1182/blood-2004-04-1439 15315974

[18] Heiligenhaus A, Steinmetz B, Lapuente R, *et al.* Recombinant tissue plasminogen activator in cases with fibrin formation after cataract surgery: a prospective randomised multicentre study. Br J Ophthalmol. 1998;82:810–5. 10.1136/bjo.82.7.810 PMC1722665 9924378

[19] Tripathi BJ, Geanon JD, Tripathi RC. Distribution of tissue plasminogen activator in human and monkey eyes. An immunohistochemical study. Ophthalmology. 1987;94:1434–8. 10.1016/s0161-6420(87)33278-6 3120076

[20] Fabbro SK, Smith SM, Dubovsky JA, Gru AA, Jones JA. Panniculitis in patients undergoing treatment with the Bruton tyrosine kinase inhibitor ibrutinib for lymphoid leukemias. JAMA Oncol. 2015;1:684–6. 10.1001/jamaoncol.2015.0457 26182170

